# Development of alkaline phosphatase-scFv and its use for one-step enzyme-linked immunosorbent assay for His-tagged protein detection

**DOI:** 10.1515/biol-2022-0521

**Published:** 2022-11-11

**Authors:** Shuzhen He, Ruixian Xu, Huashan Yi, Zhixin Chen, Congjie Chen, Qiang Li, Qinqin Han, Xueshan Xia, Yuzhu Song, Junwei Xu, Jinyang Zhang

**Affiliations:** Research Center of Molecular Medicine of Yunnan Province, Faculty of Life Science and Technology, Kunming University of Science and Technology, Kunming 650500, China; Department of Clinical Veterinary Medicine, College of Veterinary Medicine, Southwest University, Rongchang, Chongqing 402460, China

**Keywords:** alkaline phosphatase, direct ELISA, glutathione *S*-transferase, His-tagged fusion protein, one-step Western blot, scFv

## Abstract

A histidine (His)-tag is composed of six His residues and typically exerts little influence on the structure and solubility of expressed recombinant fusion proteins. Purification methods for recombinant proteins containing His-tags are relatively well-established, thus His-tags are widely used in protein recombination technology. We established a one-step enzyme-linked immunosorbent assay (ELISA) for His-tagged recombinant proteins. We analyzed variable heavy and light chains of the anti-His-tag monoclonal antibody 4C9 and used BLAST analyses to determine variable zones in light (VL) and heavy chains (VH). VH, VL, and alkaline phosphatase (ALP) regions were connected via a linker sequence and ligated into the pGEX-4T-1 expression vector. Different recombinant proteins with His tags were used to evaluate and detect ALP-scFv activity. Antigen and anti-His-scFv-ALP concentrations for direct ELISA were optimized using the checkerboard method. ZIKV-NS1, CHIKV-E2, SCRV-N, and other His-tag fusion proteins demonstrated specific reactions with anti-His-scFv-ALP, which were accurate and reproducible when the antigen concentration was 50 µg mL^−1^ and the antibody concentration was 6.25 µg mL^−1^. For competitive ELISA, we observed a good linear relationship when coating concentrations of recombinant human anti-Müllerian hormone (hAMH) were between 0.78 and 12.5 µg mL^−1^. Our direct ELISA method is simple, rapid, and accurate. The scFv antibody can be purified using a prokaryotic expression system, which provides uniform product quality and reduces variations between batches.

## 1 Introduction

The advantages of enzyme-labeled antibodies in disease diagnostics and basic research are evidenced by their broad applications. These conjugates not only provide antibody specificity but also signals can be amplified by enzymatic conversion processes [1,2,3]. Traditional chemical coupling methods are costly, often with large variations between manufactured bioconjugates. Therefore, to reduce cost, polyclonal antiserum is typically used to prepare enzyme-labeled secondary antibodies. However, additional experimental steps prolong times and increase the probability of errors.

Histidine (His)-tags are indispensable tools in many research laboratories, as typically no interactions exist between His-tags and bacterial transcription or translation machinery, so tags exert little effects on target protein characteristics [4,5,6]. The molecular weight of the His-tag is <1 kDa, unlike the maltose binding protein-tag, which is significantly larger and leads to significant changes in fusion protein solubility, and also potential problems with protein expression and purification [7,8,9]. Additionally, His-tags mainly rely on the specificity of adjacent His residues to chelate divalent metal ions during purification processes, which makes His-tagged protein-associated purification processes simple and easy [10]. Recently, Yoshimatsu et al. reported a metal-free synthetic hydrogel copolymer with affinity and selectivity for His6-tagged peptides and proteins [11]. Traditional methods are still widely applied [12,13,14,15]. Indirect enzyme-linked immunosorbent assay (ELISA) is widely used to detect His-tagged proteins, but the process is time-consuming. Therefore, simpler and faster detection techniques are imperative [16].

The single-chain variable fragment (scFv) is a synthetic antibody, which is induced and expressed in *Escherichia coli* via genetic engineering. Its heavy chain (VH) and light chain variable regions (VL) are connected by a linker. When compared with typical antibodies, scFv has a low molecular weight while retaining high affinity for corresponding antigens [17]. Fortunately, this low molecular weight facilitates easy entry into different cell compartments [18]. The appearance of scFv is due to the possibility of viral contamination and high costs limit the clinical utility of monoclonal antibodies. Through genetic engineering, recombinant enzyme-scFv fusion antibodies have been constructed to avoid issues during second antibody use. Combining the alkaline phosphatase (ALP) gene with scFv has also provided several advantages in disease diagnostics, such as convenience, high stability, and sensitivity [19,20,21,22].

The VH and VL sequences of anti-His monoclonal antibody 4C9 [23] linked to ALP were expressed in a prokaryotic system. After purification, we generated anti-His-scFv-ALP that conveniently detected His-tag-related products in one-step ELISA did not require secondary antibody binding. ELISA is widely used in different detection fields, especially the pharmaceutical industry [24,25]. When compared with indirect ELISA, the one-step procedure saves time and money [26,27,28]. Therefore, anti-His-scFv-ALP was successfully prepared and used to establish a one-step ELISA to detect His-tagged fusion proteins [29]. Our procedure facilitates the rapid detection of related products in the laboratory, provides a reference for the further rapid detection of His-tag related products, and lays the foundation for subsequent studies.

## 2 Materials and methods

### 2.1 Materials

The pGEX-4T-1 plasmid, *E. coli* Rosetta (DE3) strain, ZIKV-NS1 [30,31], CHIKV-E2, and SCRV-N [32] were stored in our laboratory. *Para*-Nitrophenylphosphate (pNPP) color development reagent was purchased from Makewonder Biotechnology Co., Ltd (Beijing, China). Glutathione *S*-transferase (GST) purification columns were purchased from Beyotime Biotechnology Co., Ltd (Shanghai, China). Other reagents were analytically pure.

### 2.2 Methods

#### 2.2.1 Identification and analysis of the variable region of a monoclonal antibody against His-tagged protein

Total RNA was extracted from 4C9 subcloned hybridoma cells using Trizol (Invitrogen, USA). First-strand cDNA was synthesized from total RNA using a reverse transcription kit (Vazyme, China). cDNAs encoding the antibody variable domains (VH and VL) were amplified by polymerase chain reaction (PCR) and cloned into the pMD-19T vector and sent to TSINGKE Biotechnology Co., Ltd (Kunming, China) for sequence analysis. Confirmed cDNA sequences were compared with nucleotide sequences at GenBank (https://www.ncbi.nlm.nih.gov/igblast/igblast.cgi).

#### 2.2.2 ALP-scFv for His-tagged fusion protein was sequenced and synthesized

**Figure 1:**
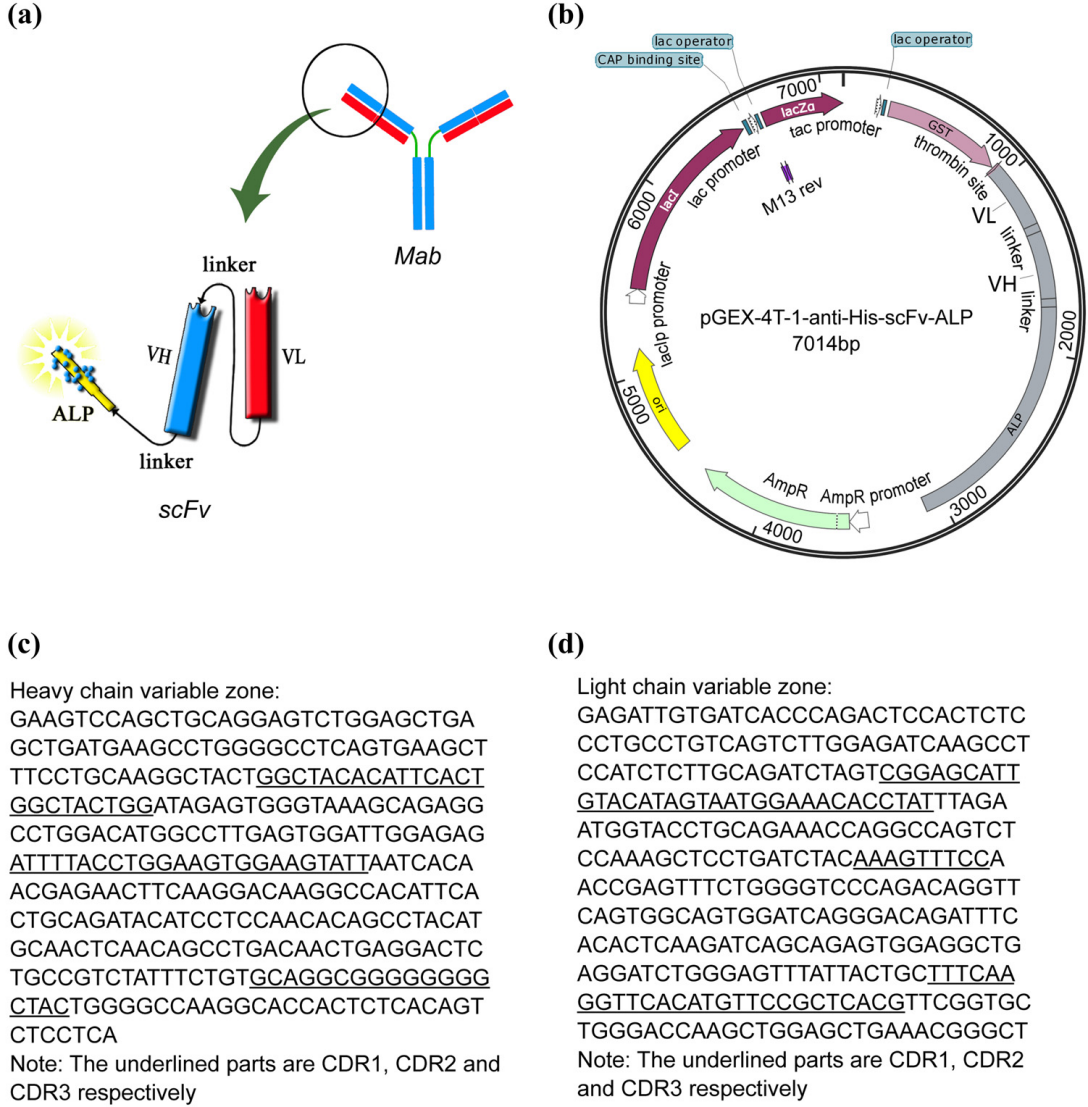
(a) Schematic showing anti-His-scFv-ALP, (b) pGEX-4T-1-anti-His-scFv-ALP, (c) heavy chain variable region sequences of the anti-His monoclonal antibody 4C9, and (d) light chain variable region sequences of the anti-His monoclonal antibody 4C9.

After total RNA extraction, a cDNA library was generated by reverse transcription. Specific primers amplifying VL (For: GAGATTGTGATCACCCAGACTCCA; Rev: GATGCTGCACCAACTGTATCC) and VH (For: GAAGTCCAGCTGCAGGAGTC; Rev: GCCAAAACGACACCCCCATCTGTCTAT) were designed and synthesized by TSINGKE Biotechnology Co., Ltd (Kunming, China). The target sequence was then amplified by PCR. Heavy and light chains were connected via a linker, which was then conjugated to ALP (Figure 1a) and cloned into the pGEX-4T-1 vector (Figure 1b).

#### 2.2.3 Optimization of anti-His-scFv-ALP expression and purification conditions

pGEX-4T-1-anti-His-scFv-ALP was transformed into *E. coli* Rosetta cells [33], which were cultured overnight (37°C at 150 rpm) in LB medium plus 100 µg mL^−1^ ampicillin. Cells were divided into 1 mL aliquots and induced with 1.0 mM isopropyl-1-thio-β-d-galactopyranoside (IPTG) for 16 h at 100 rpm at 20°C or 37°C. Then, uninduced bacteria were supplemented with 0, 0.1, 0.2, 0.3, 0.4, 0.5, and 0.6 mM IPTG for 16 h at 100 rpm at 20°C. To identify ideal induction conditions, 300 mL of LB plus ampicillin was supplemented with 1 mL of overnight bacterial culture and cultured in a shaking incubator at 37°C and 180 rpm until the OD_600nm_ reached 0.6. Anti-His-scFv-ALP fusion protein expression was induced by adding 0.5 mM IPTG at 20°C for 16 h at 100 rpm. Cells were collected by centrifugation (5,000 rpm for 10 min at 4°C) and resuspended in 40 mL of 0.01 M phosphate buffered saline (PBS) at pH 7.4. Cells were lysed by ultrasonication (200 W for 30 min), after which the lysate was centrifuged (10,000 rpm for 20 min at 4°C). The supernatant was collected and defined as the soluble protein fraction. The pellet was washed in 3 M urea (1 L 0.01 M PBS plus 3 M urea) and centrifuged for 20 min at 12,000 rpm at 4°C. The supernatant was discarded, and an appropriate volume of 1 L 0.01 M PBS plus 8 M urea was added to the pellet at 4°C until dissolution [34,35]. The volume was added to a dialysis bag, and precooled 0.01 M PBS plus 8, 6, 4, 2, and 0 M urea was used for dialysis at 4°C for 3–5 h, respectively, during which time solutions were replaced 2–3 times [36]. The supernatants and precipitates after renaturation were purified on a GST column (Beyotime, China), equilibrated in 20 mL of PBS and washed with 10 mL of elution buffer containing 20 mM Tris-HCl, 10 mM GSH, 1 mM DDT, pH 8.0 [37,38]. The anti-His-scFv-ALP conjugate was analyzed using the Bradford Protein Assay and sodium dodecyl sulfate-polyacrylamide gel electrophoresis (SDS-PAGE).

#### 2.2.4 Western blotting

Purified anti-His-scFv-ALP (GST-tag) from SDS-PAGE was transferred to a nitrocellulose membrane and non-specific sites blocked by soaking in 5% skimmed milk at 37°C for 2 h. The membrane was then washed five times in phosphate buffered saline tween-20 (PBST), incubated for 2 h with an anti-GST antibody at 37°C, washed, incubated with IgG-HRP for 1 h, and washed three times in PBST. Super enhanced chemiluminescent western blotting substrate (Biobest, Australian) was added and membrane signals observed under chemiluminescence imaging.

ZIKV-NS1 (His-tag) and JEV-NS1 (GST-tag) protein bands were similarly treated. Blots were incubated for 2 h with anti-His-scFv-ALP (10 µg mL^−1^) at 37°C, washed in PBST, and incubated with BCIP/NBT reagent (Beyotime, China) for 30 min in the dark, and signals observed directly.

#### 2.2.5 Dot blot analysis

A grid was marked on a nitrocellulose membrane to indicate the blotting region. Using a narrow-mouth pipette tip, 2 μg ZIKV-NS1 (His-tag) and CHIKV-E2 (His-tag) were spotted to determine anti-His-scFv-ALP activity, while JEV-NS1 (GST-tag) and PBS were used as negative controls. After the membrane was dry, non-specific sites were blocked by soaking membranes in PBST plus skimmed milk at 37°C for 2 h. The membrane was incubated with anti-His-scFv-ALP (10 µg mL^−1^) in 5% skimmed milk for 2 h at 37°C, washed in PBST, incubated with BCIP/NBT reagent for 30 min in the dark, and signals observed directly.

#### 2.2.6 Optimization of the direct ELISA method

Antigen and antibody concentrations were optimized using the checkerboard method. ZIKV-NS1 (His-tag), CHIKV-E2 (His-tag), and SCRV-N (His-tag) underwent a two-fold serial dilution in 50 mM carbonate coating buffer (pH 9.6) from 100 to 0.78 µg mL^−1^. Then 100 μL was added to wells in a 96-well plate and incubated at 37°C for 2 h using JEV-NS1 (GST-tag) as a negative control. The coating liquid was removed and washed with PBST for three times. PBST aliquots (200 μL) plus skimmed milk were used to block well for 2 h at 37°C. After buffer removal, purified anti-His-scFv-ALP was diluted in skimmed milk from 100 to 0.78 µg mL^−1^, after which 100 μL was added to wells and incubated at 37°C for 2 h. The antibody was removed and washed with PBST for three times. Then pNPP was added to wells and the plate incubated in the dark for 30 min, after which 50 μL of 2 N NaOH was added to quench reactions. The OD_405nm_ was determined using a microplate reader. To reduce experimental error, experiments were performed three times (*n* = 3) and the data averaged.

#### 2.2.7 Optimization of the competitive ELISA method

ZIKV-NS1 (His-tag) was diluted in 50 mM carbonate coating buffer (pH 9.6) to 10 µg mL^−1^, with 100 μL added to wells in a 96-well plate and incubated at 37°C for 2 h. JEV-NS1 (GST-tag) was used as a negative control. The coating liquid was removed and washed with PBST for three times. PBST aliquots (200 μL) plus skimmed milk were added to wells and blocking performed for 2 h at 37°C. After buffer was removed, human anti-Müllerian hormone (hAMH, His-tag) [39] was diluted from 100 to 0.78 µg mL^−1^. A 50 μL aliquot was then mixed with 50 μL anti-His-scFv-ALP (25 µg mL^−1^) and added to wells and incubated for 2 h at 37°C. Then, the solution was removed and washed with PBST for three times. pNPP was then added and the plate incubated in the dark for 30 min, after which 50 μL of 2 N NaOH was added to quench reactions. The OD_405nm_ was determined using a microplate reader. To reduce experimental error, experiments were performed three times (*n* = 3) and the data averaged.

#### 2.2.8 Statistical analysis

ELISA results underwent relative linear regression analysis in Graphpad Prism software, while one-way analysis of variance results were represented as mean ± standard deviation.

## 3 Results

### 3.1 Analysis of variable region sequences of heavy chain and light chain of anti-his monoclonal antibody

Antibody variable regions are divided into framework regions and complementarity determining regions. Our sequence analysis of VH and VL of the anti-His-tagged protein monoclonal antibody 4C9 is shown in Figure 1c and d. The antigen-binding sites of antibody 4C9 were successfully determined. Sequences were truncated as required to facilitate scFv construction and expression.

### 3.2 Optimization of anti-His-scFv-ALP expression and purification

**Figure 2:**
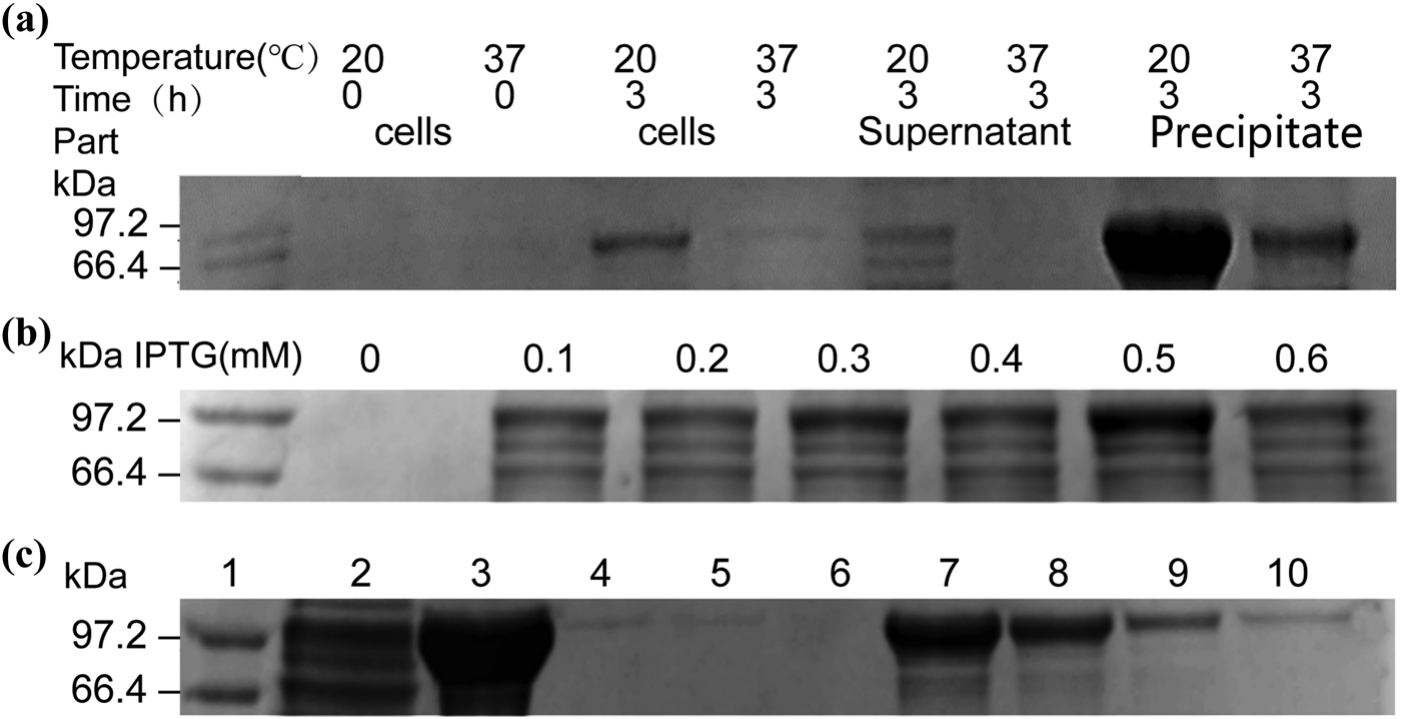
Optimization of anti-His-scFv-ALP expression. (a) Induction temperature; (b) inducer concentrations; and (c) purification results: (1) marker, (2) supernatant after ultrasonic treatment, (3) precipitate after ultrasonic treatment, (4) the first fractions of anti-His-scFv-ALP purified from supernatant, (5) the second fractions of anti-His-scFv-ALP purified from supernatant, (6) the third fractions of anti-His-scFv-ALP purified in supernatant, (7) the first fractions of anti-His-scFv-ALP purified from precipitate, (8) the second fractions of anti-His-scFv-ALP purified from precipitate, (9) the third fractions of anti-His-scFv-ALP purified from precipitate, and (10) the fourth fractions of anti-His-scFv-ALP purified from precipitate.

After comparing different temperatures (Figure 2a) and IPTG concentrations (Figure 2b), 20°C and 0.5 mM IPTG were selected. Although inclusion body purification took longer, the yield was significantly higher when compared with supernatants (Figure 2c). Ultimately, a large amount of anti-His-scFv-ALP was obtained.

### 3.3 Western blot and dot blot analyses

**Figure 3:**
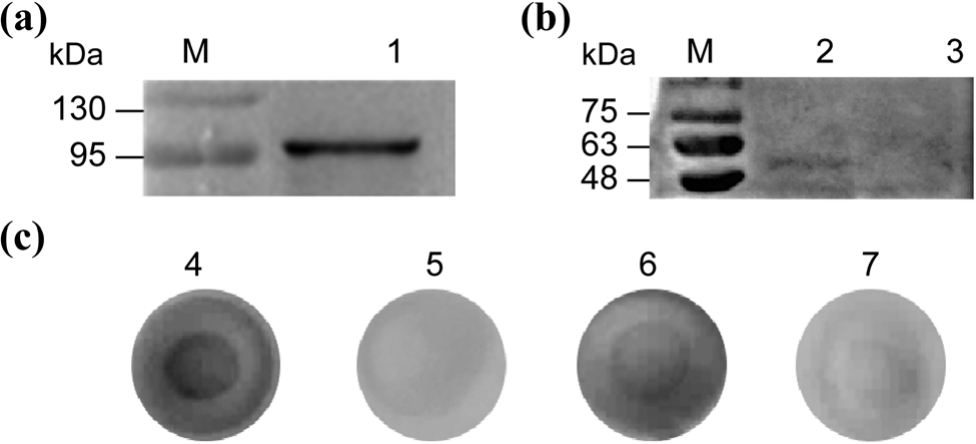
Identification and characterization of anti-His-scFv-ALP. (a) Western blot, (b) direct western blot, and (c) dot blot. M, Marker: (1) loading sample is anti-His-scFv-ALP; (2) loading sample is ZIKV-NS1 (His-tag); (3) coating antigen is JEV-NS1 (GST-tag); (4) coating antigen is ZIKV-NS1 (His-tag); (5) coating antigen is PBS; (6) coating antigen is CHIKV-E2 (His-tag); and (7) coating antigen is JEV-NS1 (GST-tag).

Western blot and dot blot are used to determine protein molecular weight and expression, especially for genetically engineered fusion proteins. Anti-GST antibody was used as the primary antibody in western blot analysis to identify anti-His-scFv-ALP (GST-tag). The target band had a molecular weight of 107 kDa, which indicated successful scFv purification (Figure 3a). ZIKV-NS1 (58 kDa, His-tag) could be observed, indicating that Anti-His-scFv-ALP is active (Figure 3b). We generated the same results by dot blot. A color reaction was determined on nitrocellulose membranes where His-tagged proteins such as ZIKV-NS1 and CHIKV-E2 were present, but nothing was observed for PBS or JEV-NS1 (Figure 3c). These results indicated that anti-His-scFv-ALP successfully bound to His-tagged proteins.

### 3.4 Determination of anti-His-scFv-ALP activity using direct ELISA

**Figure 4:**
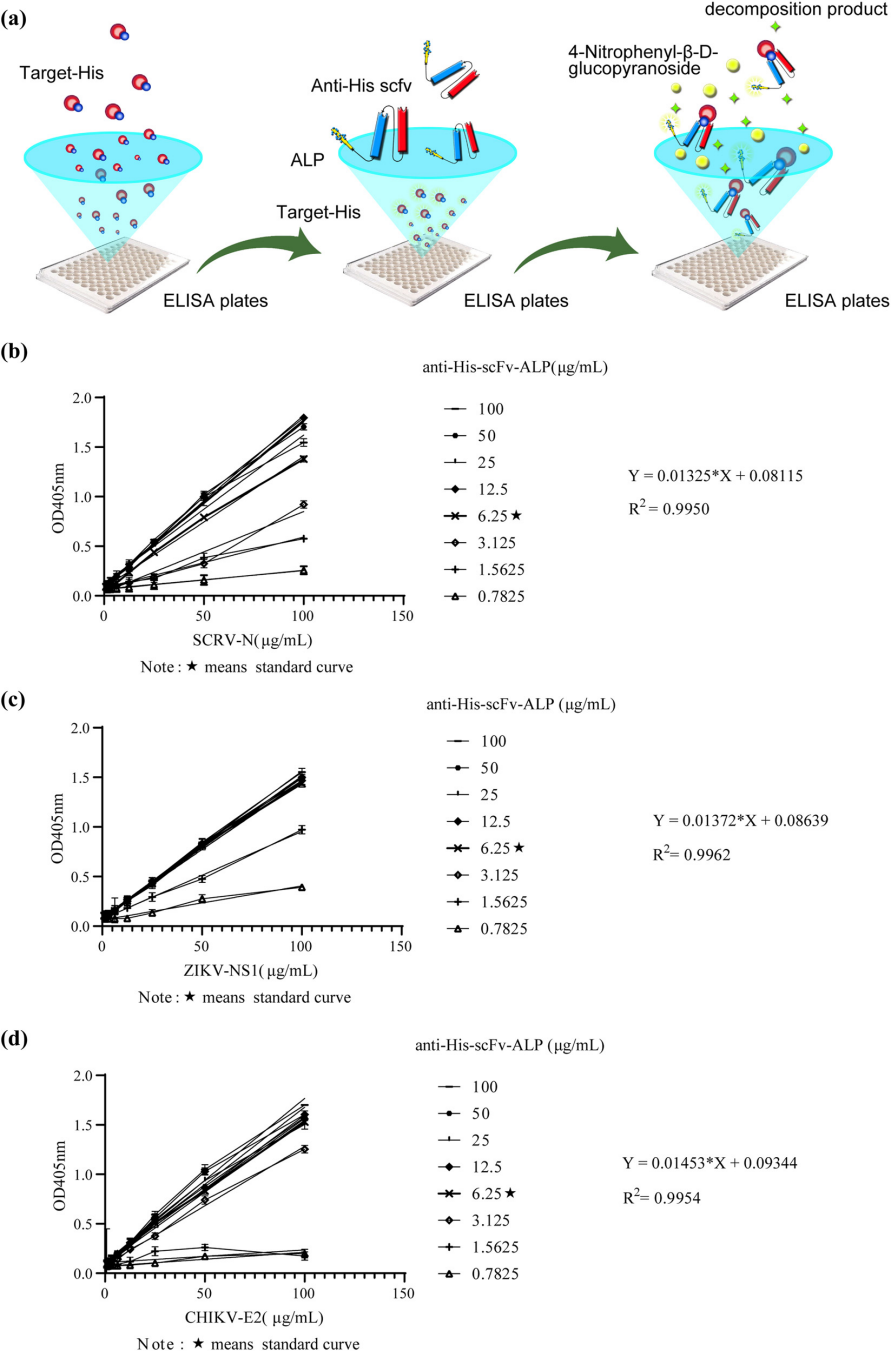
Direct anti-His-scFv-ALP ELISA. (a) Sketch map, (b) detection of His-tagged recombinant protein SCRV-N, (c) detection of His-tagged recombinant protein ZIKV-NS1, and (d) detection of His-tagged recombinant protein CHIKV-E2.

The direct ELISA principle using ALP is shown in Figure 4a. Anti-His-scFv-ALP identified His-tagged proteins such as ZIKV-NS1, CHIKV-E2, and SCRV-N, which is shown in Figure 4b–d. When the antibody concentration was 6.25 µg mL^−1^, it effectively recognized several His-tagged proteins. As concentrations increased, the OD value did not significantly change. This suggested that at 6.25 µg mL^−1^, the antibody tended to saturate the system and detection results were more stable. When the antigen concentration was 50 µg mL^−1^ and the OD value > 0.8, this facilitated His-tagged protein detection. When antigen concentration was 50 µg mL^−1^, the OD value at an antibody concentration of 6.25 µg mL^−1^ was extremely significantly different from the OD value at an antibody concentration of 0.7825 µg mL^−1^, *p* < 0.0001. The OD value at an antibody concentration of 6.25 µg mL^−1^ was compared with the OD value at an antibody concentration of 100 µg mL^−1^, *p* < 0.01. Therefore, the antibody concentration of 6.25 µg mL^−1^was chosen as the best value to ensure the experimental results and save cost. A standard curve showed an *R* ^2^ value of >0.99, indicating good linear relativity. The linear equation from the standard curve was used to measure His-tagged protein concentrations by diluting reagents within the linear range and slotting values into the equation to calculate target protein concentrations.

### 3.5 Development of a competitive ELISA method

**Figure 5:**
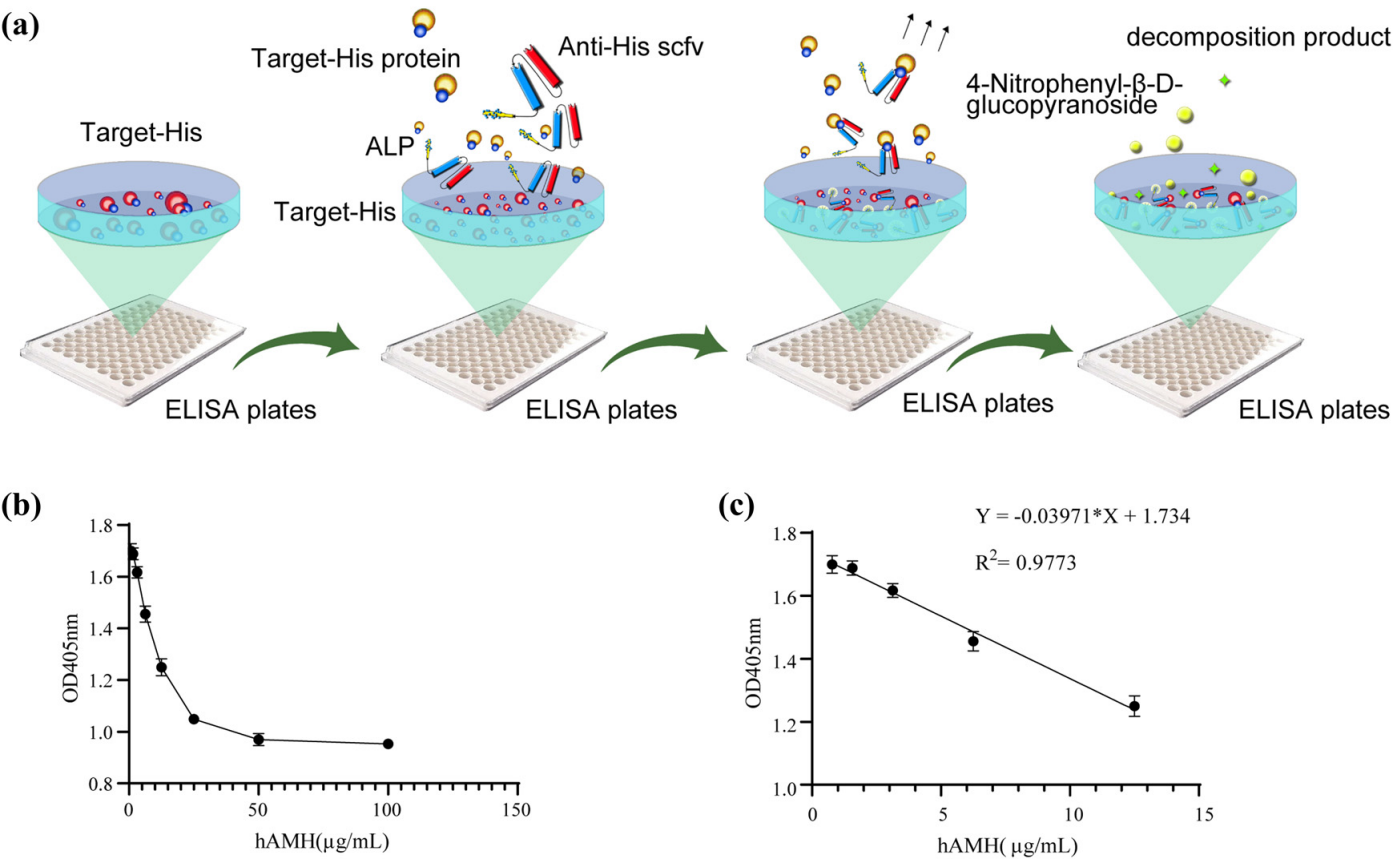
Competitive anti-His-scFv-ALP ELISA. (a) Sketch map, (b) detection of His-tagged recombinant protein hAMH, and (c) standard curve using His-tagged recombinant protein hAMH as a coating protein.

Competitive ELISA principles using ALP are shown in Figure 5a. A standard curve was constructed using hAMH concentrations on the *x*-axis and hAMH OD_405 nm_ values on the *y*-axis. When hAMH concentrations were between 0.78 and 12.5 µg mL^−1^, the absorbance had a linear relationship with concentration, with a large slope. Therefore, this region was selected for linear regression analysis (Figure 5b), with the fitting curve showing strong linearity in this region. Finally, a standard curve was constructed over this concentration range (Figure 5c); the standard curve equation was *Y* = −0.03971 × *X* + 1.734 (*R* ^2^ = 0.9773). This indicated that competitive ELISA results were more accurate when antigen concentrations were within this range.

## 4 Discussion

The pGEX-4T-1 vector expressed a GST tag with a molecular weight of 26 kDa. The molecular weight of the GST tag recombinant protein was too large to form a soluble protein. Some methods can be used to increase soluble expression, reducing protein synthesis rates, changing the medium, co-expressing proteins with molecular chaperones, and allowing proteins to unfold and refold. We showed that recombinant anti-His-scFv-ALP was induced at a low temperature, produced poorly soluble expression products in *E coli*, with purified scFv failing to meet our study requirements. Therefore, we purified scFv from inclusion bodies using gradient urea renaturation. Dilution refolding, gradient dialysis, molecular sieve chromatography, and ion-exchange chromatography can be used to facilitate protein refolding [40]. When a high urea concentration is used for cracking and refolding, fusion proteins can be easily deactivated. After denaturing in urea, we used gradient dialysis to refold the anti-His-scFv-ALP. However, some shortcomings were observed [41]. Because antibody storage at 4°C causes activity loss over time, study manipulations were performed rapidly on ice.

Due to their short operating times and low costs, we used direct and competitive ELISA to identify proteins. However, reaction conditions required optimization. The checkerboard method was used to optimize antigen coating concentrations and optimal antibody working concentrations. These steps ensured that our method is more reliable for detecting His-tagged fusion proteins. Although several traditional methods have been used to detect His-tagged fusion proteins, e.g., western blotting or indirect ELISA, new methods have been developed and include immunosensors [42] or UVHis-PAGE [43], but these are expensive and require complex instrumentation. Our antibodies could be faster and more inexpensive for biosensor applications if material costs could be reduced. ELISA is a highly accurate and rapid method; it is effective, specific, and generates virtually no matrix effects in hybrid samples. Additionally, direct ELISA does not require secondary antibodies, which not only reduces costs but also shortens detection times. One-step western blots and dot blots have the same benefits. Thus, we showed that one-step ELISA is a promising detection tool, whereas anti-His-scFv-ALP is used to detect His-tagged proteins. Although similar antibodies are available [44], no in-depth method optimization has been performed. Our remit was to establish a method for the rapid detection of His-tagged proteins.

## 5 Conclusions

Due to high anti-His 4C9 potency, single-chain antibodies were prepared and purified to generate pure antibodies with good reactivity in both direct and competitive ELISA. The *R* ^2^ value was >0.97. Our method was rapid and straightforward and provided accurate and reproducible results. We focused on the establishment of different detection methods based on purified single-chain antibodies. Commercially available antibodies require the addition of HRP secondary antibodies, while anti-His tag-scFv-ALP directly uses ALP for color development, thereby reducing experimental steps and generating quicker results. Our next step is to generate efficient kits.
